# Quantifying the heterogeneity of cognitive functioning in Alzheimer’s disease to extend the placebo-treatment dichotomy: Latent class analysis of individual-participant data from five pivotal randomized clinical trials of donepezil

**DOI:** 10.1192/j.eurpsy.2021.8

**Published:** 2021-02-15

**Authors:** Stephen Z. Levine, Yair Goldberg, Kazufumi Yoshida, Myrto Samara, Andrea Cipriani, Takeshi Iwatsubo, Stefan Leucht, Toshiaki A. Furawaka

**Affiliations:** 1 Department of Community Mental Health, University of Haifa, Haifa 3498838, Israel; 2 Faculty of Industrial Engineering and Management, Technion-Israel Institute of Technology, Haifa, Israel; 3 Department of Health Promotion and Human Behavior, Kyoto University Graduate School of Medicine / School of Public Health, Kyoto, Japan; 4 Department of Psychiatry and Psychotherapy, Technical University of Munich, School of Medicine, Munich, Germany; 5 3rd Department of Psychiatry, School of Medicine, Aristotle University of Thessaloniki, Thessaloniki, Greece; 6 Department of Psychiatry, University of Oxford, Oxford, United Kingdom; 7 Department of Neuropathology, Graduate School of Medicine, The University of Tokyo, Tokyo, Japan

**Keywords:** Clinical trials, dementia, psychometrics

## Abstract

**Background:**

The extent and profiles of heterogeneity in cognitive functioning among participants in clinical trials of antidementia medication are unknown. We aimed to quantify and identify profiles of heterogeneity of cognition in Alzheimer’s disease.

**Methods:**

Individual-level participant data were analyzed from five pivotal clinical trials of donepezil for Alzheimer’s disease (*N* = 2,919). Based on Alzheimer’s Disease Assessment Scale–Cognitive Subscale total scores from baseline up to week 12, a latent class model was used to identify heterogeneous groups. A logistic regression model was used to examine factors associated with group membership. Sensitivity analysis was conducted, restricted to the donepezil, and then the placebo arm.

**Results:**

The latent class model identified three classes labeled as low scorers (i.e., least cognitive impairment; *N* = 1,666, 76.04%), improvers (*N* = 27, 1.23%), and high scorers (*N* = 498, 22.73%). Logistic modeling showed that donepezil compared to placebo was significantly (*p* < 0.05) positively associated with membership in the improvers class (OR = 6.88, 95% CI = 2.03, 42.95), and negatively with high scorers (OR = 0.79, 95% CI = 0.64, 0.98). Sensitivity analysis restricted to the placebo, then donepezil arms replicated similar heterogeneity patterns.

**Conclusions:**

Our results inform clinicians regarding the extent of heterogeneity in cognitive functioning during treatment and contribute to trial design considerations.

## Introduction

Alzheimer’s disease is an irreversible neurodegenerative disorder characterized by progressive impairments in cognition, daily living and social functioning activities [1, 2], and ultimately death [3]. To date, five antidementia drugs have been approved by the FDA [4], and clinical trials of Alzheimer’s disease have shown small effects [5]. For over 15 years, no new antidementia drug has been approved to treat Alzheimer’s disease [6], hence most antidementia drug trials failed [7–9]. Multiple explanations exist of the preponderance of failed clinical trials of Alzheimer’s disease. [7–9] Examples include the need for longer study durations and younger participant age (i.e., under 70) to capture disease progression [10]. The role of sex differences is unclear since sex is rarely a part of efficacy analyses in clinical trials for Alzheimer’s disease [11]. Nonetheless, female sex is more common in the population of persons with Alzheimer’s disease [4], and clinical trials of Alzheimer’s disease [11]. Another explanation of failed clinical trials of Alzheimer’s disease may be that the course of cognition in Alzheimer’s disease is heterogeneous [12–16], which makes attaining research and clinical goals particularly challenging. Thus, understanding heterogeneity in Alzheimer’s disease may contribute to clinical trial design and treatment [17].

Statistical methods (e.g., latent class modeling) have been used to quantify the extent of symptom heterogeneity in various disorders [18], including Alzheimer’s disease [13–16]. These methods identify different groups (termed classes or trajectories) with distinct progressive patterns and profiles [19]. Prior study estimates show that most persons with Alzheimer’s disease assume a slow progressive pattern of cognitive decline (72% [13], 76.5% [20], 76% [21]), while few persons assume a rapid pattern of cognitive progression (4% [22], 24% [21]). Studies have identified markers of trajectory membership to detect the sources of heterogeneity. For instance, younger age was associated with assuming a trajectory of slower disease progression [21]. However, to date, no study has examined the patterns and profiles of heterogeneity in antidementia medication for Alzheimer’s disease in clinical trials.

The current study aims to empirically quantify heterogeneous groups of cognitive functioning in Alzheimer’s disease and their profiles, based on individual participant data from five randomized clinical trials of donepezil.

## Methods

### Study design

We accessed pivotal individual-level participant data of randomized controlled double-blinded trials of donepezil conducted by Eisai Co., Ltd (see eTable 1). Data. Data access was granted following the submission of an a priori analytic plan. The data were analyzed on a secure Internet cloud-based platform (http://www.clinicalstudydatarequest.com). We included trials in which participants with Alzheimer’s disease were assessed with the Alzheimer’s Disease Assessment Scale–Cognitive Subscale (ADAS-Cog; Rosen et al., 1984). Individual-level data were ascertained from participants on five randomized clinical trials with similar follow-up intervals and ADAS-Cog scores [23–27]. Institutional review boards approved each trial, and all trial participants gave written informed consent.

### Measures

#### Alzheimer’s disease assessment scale–cognitive subscale (ADAS-Cog)

The ADAS-Cog is a neuropsychological index of the severity of the cognitive symptoms of dementia, and is the gold-standard in clinical trials of Alzheimer’s disease. [28,29]. The ADAS-Cog consists of 11 tasks (word recall, word recognition, constructional praxis, orientation, naming objects and fingers, commands, ideational praxis, remembering test instruction, spoken language, word-finding, and comprehension) that include both participant-completed and observer-based assessments. ADAS-Cog total scores range from 0 to 70, with higher scores representing a more considerable cognitive impairment.

The purpose of the ADAS-Cog is to provide a comprehensive assessment of the extent of cognitive dysfunction in Alzheimer’s disease, whereas the purpose of the widely used Mini-Mental State Examination (MMSE) is to screen for cognitive impairment in the general population. Nonetheless, conversion between MMSE and ADAS-Cog total and change scores is possible (e.g., an MMSE total score of 3 converts to an ADAS-Cog total score of 64; 10–48, 20–24, and 30–6, respectively) [30]. To interpret the results, we consider a four-point difference between groups on the ADAS-Cog as clinically relevant [31]. Furthermore, meta-analysis has estimated the disease progression rate at 5.5 points per year for a patient population with a mean baseline ADAS-cog value of 25 [32].

### Statistical analysis

At step one of the analysis, we characterized the total study population. At step two, we computed latent class mixed modeling for the total study population as the primary analysis. Latent class mixed modeling consists of model identification, plotting, examining, and labeling the resultant classes. Latent class mixed modeling empirically identifies classes in the total population that may be understood as trajectories or groups. Latent class mixed modeling groups patients into classes to minimize within-group homogeneity and maximize between-group heterogeneity. Namely, the model aims for participants within the same class to resemble one another but differ from members of the other class(es).

Model identification consisted of fitting latent class mixed models for two to six classes to identify the number of classes that best fit the data. Two to six classes were fitted with the assessment week as a linear term and then fitted as a quadratic term. A linear term conceptually implies the course assumes a straight-line of cognitive impairment over time, whereas a quadratic term means that cognitive impairment over time assumes a curvilinear form. Fixed terms in the latent models were trial, sex, age, week, and treatment. Trial was set as a fixed rather than a random effect owing to software limitations. The model with the smallest Bayesian information criterion value was chosen as the most parsimonious (described in eTable 2).

Based on the most parsimonious latent class mixed model, each participant was assigned to a class based on posterior probability values. Posterior probabilities exceeding 0.7 are considered the cut-off for good classification [33]. The most parsimonious model was plotted to examine the pattern of cognitive impairment by week, and the characteristics of each class presented. At step three of the analysis, a series of binary logistic models were computed to examine the associations between the study covariates and class membership. Latent class mixed modeling was computed in R using the *hlme* function [19].

### Sensitivity analysis

We replicated the primary analysis above (except without the treatment arm in the models), restricting to the donepezil arm and then placebo arm.

## Results

### Sample characteristics

Table 1 shows that the total analytic sample consisted of 2,191 participants with ADAS-Cog assessments. The average follow-up time was 10.77 (SD = 3.34) weeks. The average participant age at baseline was 72.42 (SD = 7.46). There were 1,339 (61.11%) females, and 852 (38.89%) males. The placebo group consisted of 760 (34.69%) participants, and the donepezil 1,431 (65.31%).

**Table 1.** Sample characteristics.

Variable	Classification	Total sample	Low scorers	Improvers	High scorers
		*M*/*N* (SD/%)	*M*/*N* (SD/%)	*M*/*N* (SD/%)	*M*/*N* (SD/%)
*N*		2191	(*N* = 1,666, 76.04%)	(*N* = 27, 1.23%)	(*N* = 498, 22.73%)
Trial *N* (%)	Homma et al. [23]	268 (12.23)	217 (13.03)	3 (11.11)	48 (9.64)
	Rogers and Friedhoff [24]	156 (7.12)	112 (6.72)	2 (7.41)	42 (8.43)
	Rogers et al. [25]	480 (21.91)	359 (21.55)	9 (33.33)	112 (22.49)
	Rogers et al. [26]	472 (21.54)	345 (20.71)	3 (11.11)	124 (24.90)
	Burns et al. [27]	815 (37.20)	633 (38.00)	10 (37.04)	172 (34.54)
Sex *n* (%)	Female	1339 (61.11)	1012 (60.74)	15 (55.56)	312 (62.65)
	Male	852 (38.89)	654 (39.26)	12 (44.44)	186 (37.35)
Age		72.42 (7.46)	72.70 (7.39)	71.85 (7.55)	71.50 (7.65)
Allocation	Placebo	760 (34.69)	567 (34.03)	2 (7.41)	191 (38.35)
	Donepezil	1431 (65.31)	1099 (65.97)	25 (92.59)	307 (61.65)
Baseline	ADAS-COG (mean [SD])	25.70 (10.58)	21.07 (6.55)	42.95 (6.87)	40.25 (6.86)
Last visit score	ADAS-COG (M [SD])	24.70 (11.40)	19.61 (6.71)	26.41 (6.75)	41.64 (6.73)
Change	ADAS-COG (M [SD])	1.00 (4.93)	1.46 (4.09)	16.54 (3.77)	−1.39 (5.52)
Final visit week	week (M [SD])	10.77 (3.34)	10.84 (3.27)	11.78 (1.15)	10.50 (3.62)
Dropout (%)	Completer	1900 (86.72)	1459 (87.58)	26 (96.30)	415 (83.33)
	Dropout	291 (13.28)	207 (12.42)	1 (3.70)	83 (16.67)

Abbreviations: ADAS-COG, Alzheimer’s disease assessment scale–cognitive subscale; M, mean; SD, standard deviation.

### Latent class mixed model

**Figure 1.**
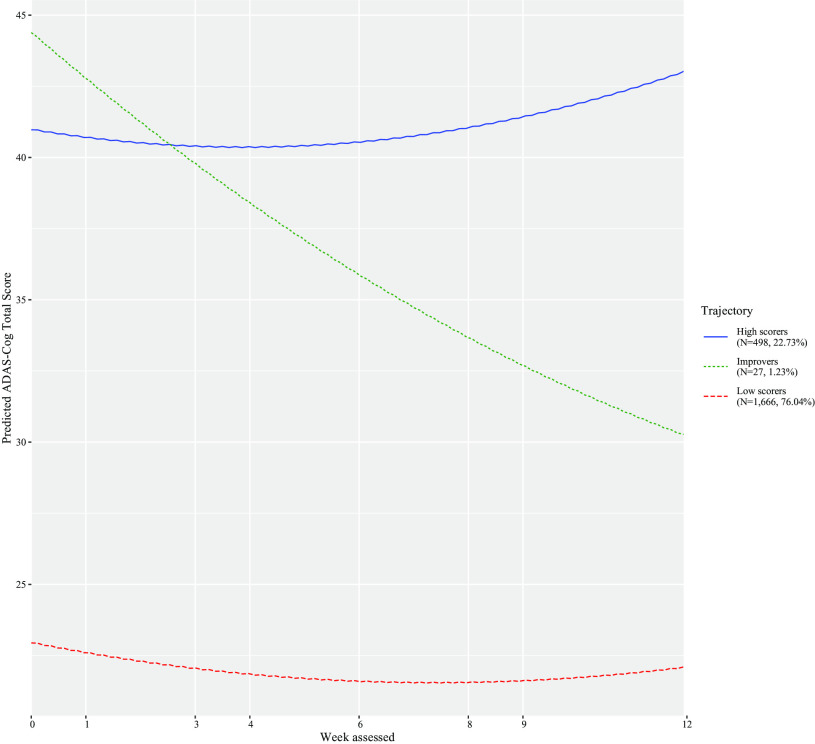
Classes identified for Alzheimer’s disease assessment scale–cognitive subscale (ADAS-Cog), their pattern by age, and the number of trial participants in each class.

The Bayesian information criterion was examined to identify the number of latent classes (see eTable 2). The best-fitting model consisted of three classes and a quadratic week term (Supplement eTable 2). Figure 1 shows that the classes consisted of trajectories of low scorers (i.e., less severe cognitive impairment; *N* = 1,666, 76.04%), improvers (*N* = 27, 1.23%), and high scorers (i.e., more severe cognitive impairment; *N* = 498, 22.73%). Table 1 shows the characteristics of each class. From baseline to the last visit, low scorers increased by approximately 1.46 ADAS-Cog points, improvers by 16.54 points, whereas high scorers dropped by −1.39 points (Table 1).

### Logistic regression modeling

Next, we used logistic regression models to predict latent class mixed model membership (eTable 3). The results showed that trial participation (except [27]) was significantly associated with low or high scorers, although unrelated to membership in the improvers class. Older age was associated with membership in the low scorer group (OR = 1.02, 95% CI = 1.01). Donepezil compared with placebo was statistically significantly associated with a greater likelihood of membership in the improvers group (OR = 6.88, 95% CI = 2.03, 42.95). Advanced age (OR = 0.98, 95% CI = 0.96, 0.99) and donepezil compared to placebo (OR = 0.79, 95% CI = 0.64, 0.98) were significantly inversely associated with a greater likelihood membership in the group of higher scorers. Consistently, sex had a null effect on class membership.

### Sensitivity analysis

**Figure 2.**
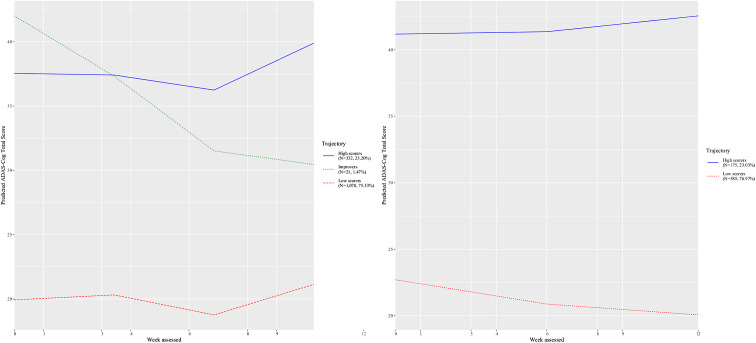
Classes identified for Alzheimer’s disease assessment scale–cognitive subscale (ADAS-Cog), their pattern by age, and the number of trial participants in each class among participants allocated to placebo and then donepezil.

We replicated the primary analysis as exactly above, but separately for patients randomized to donepezil and placebo. Based on information fit indices, the donepezil group consisted of three classes identifiable from eTable 2 as low scorers (*N* = 1,078, 75.33%), improvers (*N* = 21, 1.47%), and high scorers (*N* = 332, 23.20%). The placebo group consisted of two classes, who were low scorers (*N* = 585, 76.97%) or high scorers (*N* = 175, 23.03%) (Table 2). The class courses are shown in Figure 2.

**Table 2.** Sample characteristics by donepezil and placebo group and based on the latent class mixed model.

Variable	Classification	Donepezil				Placebo		
		Overall	Low scorers	Improvers	High scorers	Overall	Low scorers	High scorers
*N*		1431	*N* = 1,078	*N* = 21	*N* = 332	760	*N* = 585	*N* = 175
			(75.33%)	(1.47%)	(23.20%)		(76.97%)	(23.03%)
Trial *N* (%)	Homma et al. [23]	136	114	2	20	132	104	28
		(9.50)	(10.58)	(9.52)	(6.02)	(17.37)	(17.78)	(16.00)
	Rogers and Friedhoff [24]	119	86	2	31	37	27	10
		(8.32)	(7.98)	(9.52)	(9.34)	(4.87)	(4.62)	(5.71)
	Rogers et al. [25]	323	236	8	79	157	119	38
		(22.57)	(21.89)	(38.10)	(23.80)	(20.66)	(20.34)	(21.71)
	Rogers et al. [26]	310	226	1	83	162	124	38
		(21.66)	(20.96)	(4.76)	(25.00)	(21.32)	(21.20)	(21.71)
	Burns et al. [27]	543	416	8	119	272	211	61
		(37.95)	(38.59)	(38.10)	(35.84)	(35.79)	(36.07)	(34.86)
Sex *n* (%)	Female	888	664	11	213	451	345	106
		(62.05)	(61.60)	(52.38)	(64.16)	(59.34)	(58.97)	(60.57)
	Male	543	414	10	119	309	240	69
		(37.95)	(38.40)	(47.62)	(35.84)	(40.66)	(41.03)	(39.43)
Age		72.66	72.80	73.71	72.13	71.97	72.44	70.39
		(7.37)	(7.31)	(6.80)	(7.60)	(7.62)	(7.63)	(7.39)
Baseline score	ADAS-COG (M [SD])	25.50	20.77	43.86	39.68	26.08	21.68	40.81
		(10.52)	(6.42)	(7.20)	(6.71)	(10.70)	(6.97)	(7.29)
Last visit score	ADAS-COG (M [SD])	23.80	18.86	26.35	39.69	26.41	21.06	44.28
		(10.98)	(6.57)	(7.07)	(6.65)	(11.98)	(6.95)	(6.85)
Change	ADAS-Cog (M [SD])	1.70	1.92	17.51	−0.01	−0.33	0.62	−3.47
		(4.85)	(4.11)	(3.51)	(5.19)	(4.79)	(4.04)	(5.71)
Final visit week	Week (M [SD])	10.65	10.72	11.71	10.39	10.99	11.08	10.71
		(3.50)	(3.44)	(1.31)	(3.77)	(3.02)	(2.92)	(3.30)
Dropout (%)	Completer	1226	932	20	274	674	526	148
		(85.67)	(86.46)	(95.24)	(82.53)	(88.68)	(89.91)	(84.57)
	Dropout	205	146	1	58	86	59	27
		(14.33)	(13.54)	(4.76)	(17.47)	(11.32)	(10.09)	(15.43)

Abbreviations: ADAS-COG, Alzheimer’s disease assessment scale–cognitive subscale; M, mean; SD, standard deviation.

We fitted binary logistic regression models to predict class membership like the primary analysis. We restricted the analysis to the group allocated to donepezil and then placebo, and did not include the treatment term in the model (eTable 3). The trial covariate was statistically significantly (*p* < 0.05) associated with the likelihood of membership in the classes of low and high scorers in the donepezil group analysis of, but not improvers (eTable 3). Trial had null effects in the sensitivity analysis restricted to the placebo group (eTable 3). Trial had null effects in the analyses restricted to the placebo group. In the placebo analysis, advanced age was positively associated with low scorers (OR = 1.04, 95% CI = 1.02, 1.06) membership and negatively associated with high scorer membership (OR = 0.96, 95% CI = 0.94, 0.98). Age was associated with membership in the high scorers in the donepezil analysis (OR = 0.98, 95% CI = 0.97, 1.00).

## Discussion

Based on individual participant data from five randomized clinical trials of donepezil, we aimed to quantify the extent heterogeneity of cognitive impairment in Alzheimer’s disease. The results empirically identified classes of most were low scorers (*N* = 1,666, 76.04%) characterized by the worst cognitive impairment, improvers (*N* = 27, 1.23%), and high scorers (*N* = 498, 22.73%). Also, we examined markers associated with group membership.

A small group of study participants (1.23%), mostly randomized to donepezil, assumed a pattern consistent with amelioration as reflected by the clinically relevant improvement in cognition (i.e., a four-point improvement on the ADAS-COG) within 12 weeks [31]. Membership in this class was associated with donepezil rather than placebo treatment only. The lack of significant markers associated with the class of improvers suggests that concerted efforts are warranted to identify other factors associated with the likelihood of amelioration.

We interpret the results in terms of annual progression rates by converting the ADAS change scores at week 12 to annual rates by multiplying them by 52/12 (4.3). This is done to compare the observed changes in the study to estimates elsewhere [34]. Also, in practice, as the model includes a quadratic term it is difficult to extrapolate. Accordingly, annual rates will be presented here, which assume a constant annual rate of change. As seen in Table 1, the low scorer group had a change score of 1.46, which confers to a crude estimated annual rate of 6.28 (95% CI = 5.43, 7.12). This estimate falls into the range of the expected disease progression rate of 5.5 ADAS-Cog points per year [32]. This is unlike the classes of high scorers (−5.98, 95% CI = −8.06, −3.89). Over 12 weeks, the improvers group had an average change score of 16.54 (95% CI = 15.12, 17.96). It is unlikely that such a rapid change extends to a year, but considerable improvement may occur for a subgroup in the population, which warrants future research. Hence the point estimates for the high scorers and improvers are inconsistent with the standard Alzheimer’s disease progression model [32]. This albeit crude interpretation underscores the importance of understanding heterogeneity in Alzheimer’s Disease.

The results showed that select profiles were associated with group membership. In the primary analysis, the trial was associated with the low scorer and high scorer classes. However, in sensitivity analysis, this effect was replicated in the donepezil and not placebo group. This suggests that across trials, heterogeneity is a challenge to the treatment and less the placebo arm [35]. Similarly, younger age was associated with membership in the higher scorer class in the primary analysis and analysis restricted to the donepezil group, but not the placebo group. These results are in-line with prior research on age [10]. Hence the results illustrate age and trial play a role in heterogeneity.

Sex had a null association with class membership across all models. This is consistent with prior observations that sex appears not to play a role in the efficacy of Alzheimer’s Disease [11]. Nonetheless, because of the sex distribution in Alzheimer’s disease [4], further consideration of this issue is warranted.

### Limitations and conclusions

There are several limitations to our study. First, as the results are based on clinical trial data with inclusion criteria, they may have restricted generalizability. Evidence indicates that clinical trial selection criteria restrict generalizations from clinical trial data to the general population [36,37]. Accordingly, caution is warranted regarding the generalizability of the current results to clinical treatment settings. To inform clinical practice, replicating the results in large-scale naturalistic studies with more extended periods of observation may be appropriate. Second, the trials had unequal assessment intervals and were not designed to assess heterogeneity in the trajectories of long-term cognitive decline (eTable 1). Had they been, possibly different results would have been forthcoming. Third, some factors could be associated with the profiles beyond those we examined (e.g., years of education). Unfortunately, the data common to all the trials did not contain such other information. Hence, our study suffers from residual confounding, and future research may wish to examine more potential predictors of heterogeneity. Our results are restricted to donepezil and placebo. Research is warranted to examine the generalizability of these findings to other antidementia drugs. Fourth, the study duration was restricted to 12 weeks of follow-up. Given the course of cognitive decline in Alzheimer’s disease, research is warranted with longer study durations.

Fifth, we accounted for the trial as a covariate in the statistical analyses since the study data came from five randomized clinical trials. The trials had different visit schedules, follow-up intervals, and selection criteria (eTable 1); hence consideration is warranted regarding trial design [23–27]. The trial covariate was statistically significantly associated with membership in the high and low but not improvement class (eTable 3). Hence, although the trial was accounted for as a covariate, and using more trials means more variability, increasing generalizability, consideration is warranted given our use of multiple trial designs.

Sixth, we used latent class analysis to identify heterogeneity in the course of cognition. The purpose of using this method was to scrutinize how trajectories in cognition unfold with time. Alternative statistical approaches, which do not examine how heterogeneity in cognition unfolds over time, such as machine learning, hold great potency for identifying subgroups in Alzheimer’s disease [38]. Seventh, multiple other sensitivity analyses could have been computed. For example, had the analysis been conducted sequentially by trial, we would likely introduce excess type II error. In addition, the improvers were a small subgroup and would likely not be uncovered in an analysis by trial. Instead, the improvers were uncovered in the analysis of the donepezil and not placebo. In sum, the large sample based on five trials afforded us the ability to uncover a heterogeneity source in the form of an otherwise hidden subgroup.

Eight, the class of improvers in the results is small, which limits the clinical impact of our results. It is, however, not uncommon or negligible that small groups have disproportional impacts. Many examples exist of when a small segment of the population has a disproportional impact. These include the disproportionately high global burden of schizophrenia [39], and evidence that 80% of the health burden is attributable to 20% of cases [40].

Among the strengths of the current study design are five pivotal clinical trials and many participants, making the results robust. This feature reinforces our faith in the robustness of the analysis. Clinically, the results identify three courses in Alzheimer’s Disease based on ADAS-Cog scores over 12 weeks. Low scorers (76.04%) whose rate of ADAS-Cog progressive decline resembles the average rate of decline and who are characterized by placebo treatment and younger age, improvers (1.23%) who had a marked ADAS-Cog amelioration, and high scorers (22.73%) characterized by advanced age. Clinical trial and age were associated with class membership in the donepezil arm. This suggests that clinical trial designs of Alzheimer’s Disease may be required to reduce trial heterogeneity by being more targeted, at the expense of generalizability. Based on a state-of-the-art statistical analysis of five pivotal clinical trials of Donepezil for Alzheimer’s disease, the current study contributes to the literature by documenting the extent and profiles of heterogeneity in Alzheimer’s Disease under placebo or donepezil for up to 24 weeks.

## Data Availability

Data are available based on a request to http://www.clinicalstudydatarequest.com.

## Supplementary material

For supplementary material accompanying this paper visit http://dx.doi.org/10.1192/j.eurpsy.2021.8. click here to view supplementary material
