# Supplementary material for: Quantifying the heterogeneity of cognitive functioning in Alzheimer’s disease to extend the placebo-treatment dichotomy: Latent class analysis of individual-participant data from five pivotal randomized clinical trials of donepezil
Source: Eur Psychiatry. 2021 Feb 15;64(1):e16. doi: 10.1192/j.eurpsy.2021.8 (PMC8057455; doi:10.1192/j.eurpsy.2021.8)
Supplement: Supplementary file 1 [file S0924933821000080sup001.docx]

Supplement to Levine et al. Quantifying the Heterogeneity of Cognitive Functioning in Alzheimer’s Disease to extend the Placebo-Treatment Dichotomy: Latent class analysis of Individual-Participant Data from Five Pivotal Randomized Clinical Trials of Donepezil

Contents

[eTable 1 Inclusion criteria by trial in the analytic sample 2](#_Toc63265042)

[eTable 2 Supplement model fit indices 3](#_Toc63265043)

[eTable 3 Logistic regression models 4](#_Toc63265044)

[eReferences 5](#_Toc63265045)

# eTable 1 Inclusion criteria by trial in the analytic sample

| Trial | Allocation, sample, and dose information | Analytic sample  N(%) | List of visits with a scheduled ADAS-Cog administration | Trial inclusion criteria |
| --- | --- | --- | --- | --- |
| Homma et al. (2000) | Placebo 132  Donepezil 136 (5 mg) | 268 (12.23) | Week 0, 4, 8, 12, 16, 20, 24 | MMSE: 10-26 CDR: 1 or 2  ADAS-cog: at least 15 |
| Rogers and Friedhoff (1996) | Placebo 37  Donepezil 42 (1 mg)  Donepezil 40 (3 mg)  Donepezil 37 (5 mg) | 156 ( 7.12) | Week 0, 1, 3, 6, 9, 12 | MMSE: 10-26 CDR: 1 or 2 |
| Rogers, Doody, Mohs, and Friedhoff (1998) | Placebo 157  Donepezil 160 (5 mg)  Donepezil 163 (10 mg) | 480 (21.91) | Week 0, 3, 6, 9, 12 | MMSE: 10-26 CDR: 1 or 2 |
| Rogers, Farlow, Doody, Mohs, and Friedhoff (1998) | Placebo 162  Donepezil 154 (5 mg)  Donepezil 156 (10 mg) | 472 (21.54) | Week 0, 6, 12, 18, 24 | MMSE: 10-26 CDR: 1 or 2 |
| Burns et al. (1999) | Placebo 272  Donepezil 271 (5 mg)  Donepezil 272 (10 mg) | 815 (37.20) | Week 0, 6, 12, 18, 24 | MMSE: 10-26  CDR: 1 or 2 |

Abbreviations: ADAS-cog, Alzheimer's Disease Assessment Scale-Cognitive Subscale, CDR, Clinical Dementia Rating, MMSE: Mini-Mental State Examination.

# eTable 2 Supplement model fit indices

| Basis for model | Number of classes  Polynomial | BIC | SABIC | Entropy | AIC | Loglik | NPM | Largest  class |
| --- | --- | --- | --- | --- | --- | --- | --- | --- |
| Primary analysis | 2 Linear | 47172.42 | 47121.59 | 0.77 | 47081.35 | -23524.67 | 16 | 77.23 |
|  | 2 Quadratic | 46988.00 | 46921.28 | 0.77 | 46868.47 | -23413.23 | 21 | 77.23 |
|  | 3 Linear | 47068.07 | 47007.71 | 0.84 | 46959.92 | -23460.96 | 19 | 76.22 |
|  | 3 Quadratic | 46891.99 | 46812.56 | 0.84 | 46749.69 | -23349.84 | 25 | 76.04 |
|  | 4 Linear | 47017.21 | 46947.31 | 0.79 | 46891.98 | -23423.99 | 22 | 61.94 |
|  | 4 Quadratic | 46813.02 | 46720.88 | 0.87 | 46647.94 | -23294.97 | 29 | 75.99 |
|  | 5 Linear | 46953.37 | 46873.94 | 0.81 | 46811.06 | -23380.53 | 25 | 62.21 |
|  | 5 Quadratic | 46809.29 | 46704.45 | 0.84 | 46621.45 | -23277.73 | 33 | 75.49 |
|  | 6 Linear | 46955.55 | 46866.59 | 0.82 | 46796.17 | -23370.09 | 28 | 62.07 |
|  | 6 Quadratic | 46721.52 | 46603.97 | 0.85 | 46510.91 | -23218.46 | 37 | 75.22 |
| Treatment group | 2 Linear | 30723.85 | 30676.20 | 0.75 | 30644.86 | -15307.43 | 15 | 76.17 |
|  | 2 Quadratic | 30596.41 | 30532.87 | 0.76 | 30491.08 | -15225.54 | 20 | 75.33 |
|  | 3 Linear | 30637.80 | 30580.62 | 0.83 | 30543.01 | -15253.50 | 18 | 75.26 |
|  | 3 Quadratic | 30518.81 | 30442.57 | 0.83 | 30392.42 | -15172.21 | 24 | 75.33 |
|  | 4 Linear | 30612.72 | 30546.01 | 0.84 | 30502.13 | -15230.07 | 21 | 75.96 |
|  | 4 Quadratic | 30478.48 | 30389.53 | 0.85 | 30331.03 | -15137.51 | 28 | 75.40 |
|  | 5 Linear | 30588.68 | 30512.44 | 0.81 | 30462.29 | -15207.15 | 24 | 62.61 |
|  | 5 Quadratic | 30463.84 | 30362.19 | 0.86 | 30295.33 | -15115.66 | 32 | 74.63 |
|  | 6 Linear | 30600.14 | 30514.37 | 0.77 | 30457.95 | -15201.98 | 27 | 61.84 |
|  | 6 Quadratic | 30457.31 | 30342.95 | 0.78 | 30267.73 | -15097.86 | 36 | 64.85 |
| Placebo group | 2 Linear | 16415.26 | 16367.63 | 0.81 | 16345.76 | -8157.88 | 15 | 76.58 |
|  | 2 Quadratic | 16372.50 | 16308.99 | 0.81 | 16279.83 | -8119.92 | 20 | 76.97 |
|  | 3 Linear | 16409.64 | 16352.48 | 0.74 | 16326.24 | -8145.12 | 18 | 60.39 |
|  | 3 Quadratic | 16289.55 | 16213.34 | 0.88 | 16178.35 | -8065.18 | 24 | 76.32 |
|  | 4 Linear | 16394.35 | 16327.67 | 0.78 | 16297.05 | -8127.53 | 21 | 61.71 |
|  | 4 Quadratic | 16282.07 | 16193.16 | 0.85 | 16152.33 | -8048.17 | 28 | 75.00 |
|  | 5 Linear | 16356.62 | 16280.41 | 0.80 | 16245.42 | -8098.71 | 24 | 62.37 |
|  | 5 Quadratic | 16306.13 | 16204.52 | 0.87 | 16157.87 | -8046.93 | 32 | 75.00 |
|  | 6 Linear | 16397.45 | 16311.71 | 0.78 | 16272.35 | -8109.18 | 27 | 41.18 |
|  | 6 Quadratic | 16303.51 | 16189.19 | 0.83 | 16136.71 | -8032.35 | 36 | 63.55 |

Abbreviations. BIC, Bayesian information criterion, the model with the smallest BIC value is considered the most parsimonious (Schwarz, 1978). SABIC, sample-size-adjusted BIC. Entropy, higher values imply a clearer differentiation between classes (Celeux & Soromenho, 1996). AIC Akaike information criterion. AIC estimates the quality of each model, relative to each of the other models (Akaike, 1974). We relied primarily on the BIC because it is the most common index and based on prior research (Nylund, Asparouhov, & Muthén, 2007).

# eTable 3 Logistic regression models

| Analysis | Low scorers | Low scorers  (i.e., less severe cognitive impairment) | Improvers | High scorers  (i.e. more severe cognitive impairment) |
| --- | --- | --- | --- | --- |
|  | Term | OR (95% CI), P-Value | OR (95% CI), P-Value | OR (95% CI), P-Value |
| Primary analysis | Intercept | 0.68 (0.25, 1.85), 0.45 | 0.01 (0.00, 0.34), 0.01 | 1.43 (0.52, 3.95), 0.49 |
|  | Rogers & Friedhoff (1996) | **0.55 (0.35, 0.88)**, 0.01 | 0.83 (0.11, 5.14), 0.84 | **1.88 (1.16, 3.03)**, 0.01 |
|  | Rogers, et al. (1998a) | **0.62 (0.43, 0.90)**, 0.01 | 1.41 (0.41, 6.46), 0.62 | **1.59 (1.09, 2.34)**, 0.02 |
|  | Rogers, et al. (1998b) | **0.58 (0.40, 0.83)**, 0.00 | 0.47 (0.09, 2.60), 0.37 | **1.84 (1.26, 2.70)**, 0.00 |
|  | Burns et al. (1999) | 0.77 (0.54, 1.09), 0.14 | 0.88 (0.26, 3.96), 0.84 | 1.32 (0.93, 1.91), 0.13 |
|  | Male vs. ref. Female sex | 1.10 (0.90, 1.36), 0.34 | 1.31 (0.59, 2.82), 0.50 | 0.89 (0.72, 1.09), 0.25 |
|  | Age | **1.02 (1.01, 1.04)**, 0.00 | 0.99 (0.94, 1.04), 0.62 | **0.98 (0.96, 0.99)**, 0.00 |
|  | Donepezil vs. ref. Placebo | 1.15 (0.93, 1.42), 0.18 | **6.88 (2.03, 42.95)**, 0.01 | **0.79 (0.64, 0.98)**, 0.03 |
| Donepezil analysis | Intercept | 1.76 (0.50, 6.24), 0.38 | 0.00 (0.00, 0.21), 0.01 | 0.62 (0.17, 2.23), 0.46 |
|  | Rogers & Friedhoff (1996) | **0.49 (0.26, 0.89)**, 0.02 | 1.07 (0.13, 9.10), 0.94 | **2.13 (1.14, 4.05)**, 0.02 |
|  | Rogers, et al. (1998a) | **0.50 (0.29, 0.83)**, 0.01 | 1.56 (0.38, 10.50), 0.58 | **2.00 (1.18, 3.51)**, 0.01 |
|  | Rogers, et al. (1998b) | **0.49 (0.29, 0.82)**, 0.01 | 0.20 (0.01, 2.09), 0.19 | **2.27 (1.34, 3.98)**, 0.00 |
|  | Burns et al. (1999) | 0.62 (0.37, 1.00), 0.06 | 0.94 (0.23, 6.28), 0.94 | **1.68 (1.02, 2.90)**, 0.05 |
|  | Male vs. ref. Female sex | 1.11 (0.86, 1.43), 0.42 | 1.56 (0.64, 3.75), 0.31 | 0.86 (0.67, 1.11), 0.26 |
|  | Age | 1.02 (1.00, 1.03), 0.08 | 1.02 (0.96, 1.09), 0.46 | **0.98 (0.97, 1.00)**, 0.04 |
| Placebo analysis | Intercept | 0.25 (0.05, 1.32), 0.10 |  | 3.95 (0.76, 20.82), 0.10 |
|  | Rogers & Friedhoff (1996) | 0.72 (0.31, 1.73), 0.44 |  | 1.39 (0.58, 3.18), 0.44 |
|  | Rogers, et al. (1998a) | 0.73 (0.41, 1.28), 0.27 | N/A | 1.37 (0.78, 2.43), 0.27 |
|  | Rogers, et al. (1998b) | 0.80 (0.45, 1.39), 0.43 |  | 1.26 (0.72, 2.21), 0.43 |
|  | Burns et al. (1999) | 0.89 (0.53, 1.47), 0.66 |  | 1.12 (0.68, 1.89), 0.66 |
|  | Male vs. ref. Female sex | 1.10 (0.78, 1.56), 0.60 |  | 0.91 (0.64, 1.29), 0.60 |
|  | Age | **1.04 (1.02, 1.06)**, 0.00 |  | **0.96 (0.94, 0.98)**, 0.00 |

Note. Statistically significant (P<.05) values in bold for readability.

# eReferences

Akaike, H. (1974). A new look at the statistical model identification. *IEEE Transactions on Automatic Control, 19*(6), 716-723. doi:10.1109/TAC.1974.1100705

Burns, A., Rossor, M., Hecker, J., Gauthier, S., Petit, H., Moller, H. J., . . . Friedhoff, L. T. (1999). The effects of donepezil in Alzheimer's disease - results from a multinational trial. *Dement Geriatr Cogn Disord, 10*(3), 237-244. doi:10.1159/000017126

Celeux, G., & Soromenho, G. (1996). An entropy criterion for assessing the number of clusters in a mixture model. *Journal of Classification, 13*(2), 195-212. doi:10.1007/BF01246098

Homma, A., Takeda, M., Imai, Y., Udaka, F., Hasegawa, K., Kameyama, M., & Nishimura, T. (2000). Clinical efficacy and safety of donepezil on cognitive and global function in patients with Alzheimer's disease. A 24-week, multicenter, double-blind, placebo-controlled study in Japan. E2020 Study Group. *Dement Geriatr Cogn Disord, 11*(6), 299-313. doi:10.1159/000017259

Nylund, K. L., Asparouhov, T., & Muthén, B. O. (2007). Deciding on the Number of Classes in Latent Class Analysis and Growth Mixture Modeling: A Monte Carlo Simulation Study. *Structural Equation Modeling: A Multidisciplinary Journal, 14*(4), 535-569. doi:10.1080/10705510701575396

Rogers, S. L., Doody, R. S., Mohs, R. C., & Friedhoff, L. T. (1998). Donepezil improves cognition and global function in Alzheimer disease: a 15-week, double-blind, placebo-controlled study. Donepezil Study Group. *Arch Intern Med, 158*(9), 1021-1031. doi:10.1001/archinte.158.9.1021

Rogers, S. L., Farlow, M. R., Doody, R. S., Mohs, R., & Friedhoff, L. T. (1998). A 24-week, double-blind, placebo-controlled trial of donepezil in patients with Alzheimer's disease. Donepezil Study Group. *Neurology, 50*(1), 136-145. doi:10.1212/wnl.50.1.136

Rogers, S. L., & Friedhoff, L. T. (1996). The efficacy and safety of donepezil in patients with Alzheimer's disease: results of a US Multicentre, Randomized, Double-Blind, Placebo-Controlled Trial. The Donepezil Study Group. *Dementia, 7*(6), 293-303. doi:10.1159/000106895

Schwarz, G. (1978). Estimating the Dimension of a Model. *Ann. Statist., 6*(2), 461-464. doi:10.1214/aos/1176344136
